# Hyperspectral imaging facilitating resect‐and‐discard strategy through artificial intelligence‐assisted diagnosis of colorectal polyps: A pilot study

**DOI:** 10.1002/cam4.70195

**Published:** 2024-09-25

**Authors:** Cheng Peng, Chong Xuan Tian, Yijun Mu, Mingjun Ma, Zhenlei Zhang, Meng Wan, Jing Liu, Zhen Li, Xiuli Zuo, Wei Li, Yanqing Li

**Affiliations:** ^1^ Department of Gastroenterology, Qilu Hospital, Cheeloo College of Medicine Shandong University Jinan China; ^2^ Department of Biomedical Engineering Institute, School of Control Science and Engineering Shandong University Jinan China; ^3^ Laboratory of Translational Gastroenterology, Qilu Hospital, Cheeloo College of Medicine Shandong University Jinan China; ^4^ Robot Engineering Laboratory for Precise Diagnosis and Therapy of GI Tumor, Qilu Hospital, Cheeloo College of Medicine Shandong University Jinan China; ^5^ Shandong Provincial Clinical Research Center for Digestive Disease Jinan China

**Keywords:** artificial intelligence, colonoscopy, colorectal polyp, hyperspectral, imaging

## Abstract

**Background and Aims:**

The resect‐and‐discard strategy for colorectal polyps based on accurate optical diagnosis remains challenges. Our aim was to investigate the feasibility of hyperspectral imaging (HSI) for identifying colorectal polyp properties and diagnosis of colorectal cancer in fresh tissues during colonoscopy.

**Methods:**

144,900 two dimensional images generated from 161 hyperspectral images of colorectal polyp tissues were prospectively obtained from patients undergoing colonoscopy. A residual neural network model was trained with transfer learning to automatically differentiate colorectal polyps, validated by histopathologic diagnosis. The diagnostic performances of the HSI‐AI model and endoscopists were calculated respectively, and the auxiliary efficiency of the model was evaluated after a 2‐week interval.

**Results:**

Quantitative HSI revealed histological differences in colorectal polyps. The HSI‐AI model showed considerable efficacy in differentiating nonneoplastic polyps, non‐advanced adenomas, and advanced neoplasia in vitro, with sensitivities of 96.0%, 94.0%, and 99.0% and specificities of 99.0%, 99.0%, and 96.5%, respectively. With the assistance of the model, the median negative predictive value of neoplastic polyps increased from 50.0% to 88.2% (*p* = 0.013) in novices.

**Conclusion:**

This study demonstrated the feasibility of using HSI as a diagnostic tool to differentiate neoplastic colorectal polyps in vitro and the potential of AI‐assisted diagnosis synchronized with colonoscopy. The tool may improve the diagnostic performance of novices and facilitate the application of resect‐and‐discard strategy to decrease the cost.

## Graphical abstract

Considering the resect‐and‐discard strategy for colorectal polyps based on accurate optical diagnosis remains challenge, we developed and validated a tissue classification algorithm to accurately distinguish neoplastic lesions from small or diminutive colorectal polyps based on HSI data acquired from fresh colon tissues. HSI system with highly diagnostic performance of colorectal polyps could facilitate the application of resect‐and‐discard strategies, which may reduce the burden and cost of excessive pathological evaluation.

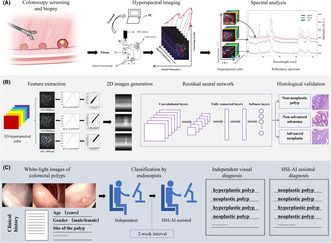

## 1 INTRODUCTION

Colorectal cancer (CRC) is the second most common cause of cancer deaths worldwide.^1^ Early detection and removal of precancerous polyps by colonoscopy is the preferred choice for preventing advanced CRC and reducing mortality.^2^, ^3^, ^4^ The majority of polyps detected during colonoscopy are small (6–9 mm) or diminutive (1–5 mm) in size, of which advanced pathological features and cancer occur infrequently.^5^

To reduce the cost related to excessive histological assessment for these low‐risk polyps, a “resect‐and‐discard strategy” based on accurate endoscopic diagnosis was proposed.^6^, ^7^ The Preservation and Incorporation of Valuable Endoscopic Innovations (PIVI) of American Society for Gastrointestinal Endoscopy (ASGE) suggested a ≥90% negative predictive value (NPV) for optically diagnosed of neoplastic polyps and a ≥90% agreement with surveillance interval recommendations of pathologic assessment.^8^ In practice, nevertheless, resect‐and‐discard strategy present a challenge to the endoscopists, for precise endoscopic prediction of small and diminutive polyp histology to differentiate the neoplastic from nonneoplastic polyps (NNP) is difficult.^9^, ^10^

In this regard, several advanced imaging techniques, referred to as optical biopsy, have been developed to derive more optical information from endoscopic images and predict polyp histology.^6^, ^11^ The narrowband imaging classification showed considerable potential to distinguish adenomas and hyperplastic polyps.^9^, ^12^ However, these methods require experienced endoscopists with sufficient training while it is difficult for community‐based physicians to obtain sufficient diagnostic abilities.^13^, ^14^ A prospective study showed that only 25% of gastroenterologists achieved ≥90% accuracy in assessing polyps using optical biopsy with narrow‐band imaging.^14^, ^15^ Although artificial intelligence (AI) was introduced to reduce the interobserver variation, the limited specificity, uninterpretability, and substantial heterogeneity among different training data limited its clinical practice.^16^, ^17^, ^18^, ^19^

Therefore, new advanced optical techniques combined with AI may improve the immediate and accurate diagnosis of polyp properties, which is essential for the promotion of resect‐and‐discard strategies. Hyperspectral imaging (HSI) is a promising new approach for the prediction of polyp histology that eliminates these drawbacks. HSI technology integrates digital imaging and spectroscopy, allowing for the extraction of morphological information and electromagnetic radiation.^20^ As a tool to aid visualization or a complementary source of information, HSI has been used in the detection and identification of neoplasia.^21^, ^22^, ^23^

In this study, we aimed to develop and validate a tissue classification algorithm to accurately distinguish neoplastic lesions from small or diminutive polyps based on HSI data acquired from fresh colorectal tissues, which was expected to facilitate resect‐and‐discard strategy.

## 2 MATERIALS AND METHODS

### 2.1 Patients and specimen preparation

This prospective diagnostic study was approved by the Ethics Committee of Qilu Hospital, Shandong University and registered at clinicalTrials.gov. (No. NCT05576506). Samples were prospectively collected from colorectal polyps in patients aged 18–75 years undergoing colonoscopy at the Endoscopy Center of Qilu Hospital, Shandong University, between October 2022 and December 2022. Patients with the followings were excluded: (i) severe physical diseases that prevented them from adhering to the examination requirements, (ii) coagulopathy or other contraindications for biopsy or polypectomy, (iii) a history of gastrointestinal surgery, (iv) presence of inflammatory bowel disease or hereditary polyposis syndromes (such as family adenomatous polyposis or Lynch syndrome) and (v) inability to provide informed consent or refusal to participate in the study.

**FIGURE 1:**
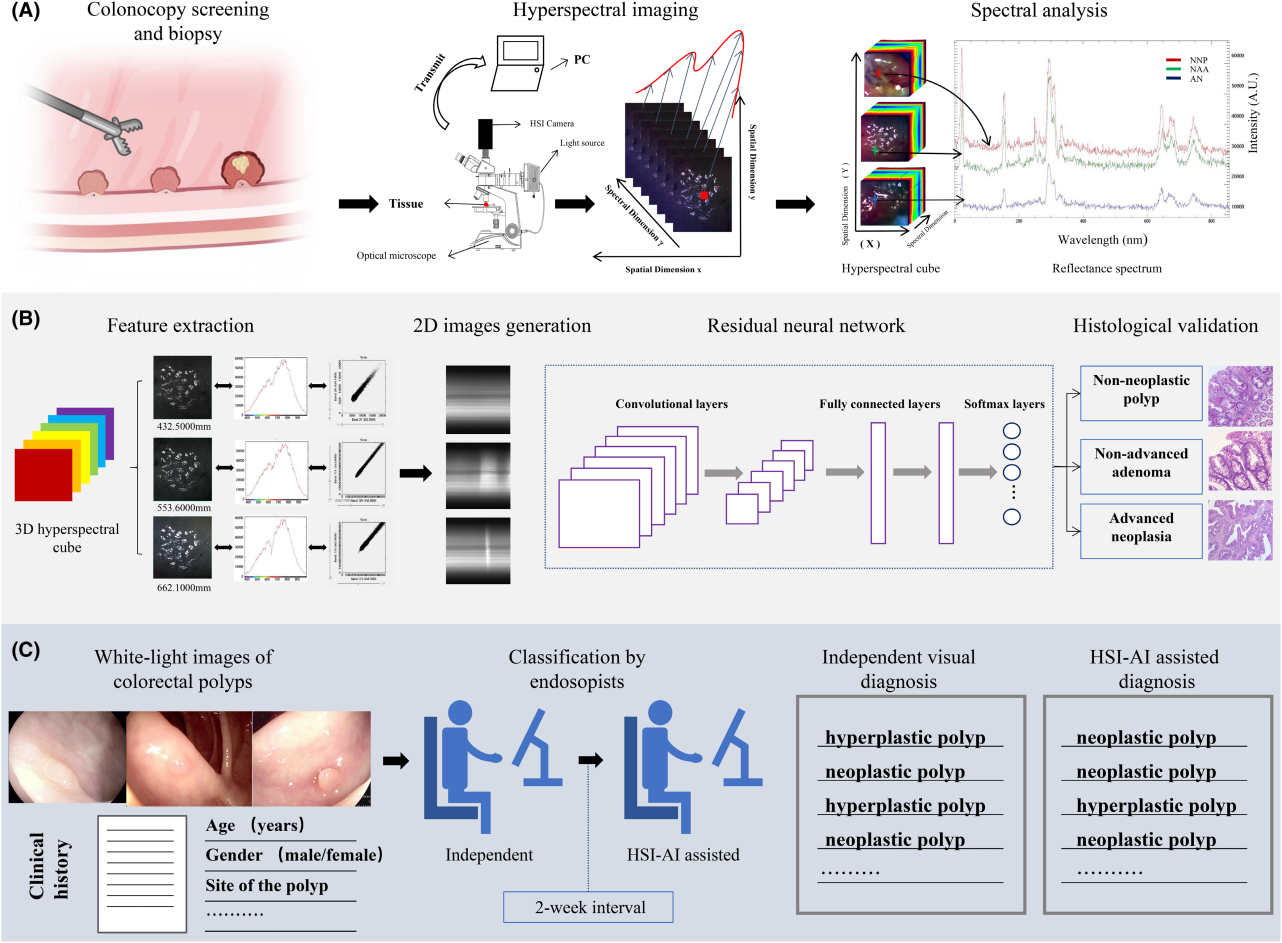
Experimental design and workflow. (A) Illustration of taking fresh tissues during colonoscopy for direct hyperspectral imaging and spectral analysis for variable tissues of polyps. (B) Spectral and spatial properties of hyperspectral cube, conversion from hyperspectral cubes to 2D images, and schematic of the residual neural network model. (C). Assessment of optical diagnosis in endoscopists with white‐light images and HSI‐AI assisted diagnosis after 2 weeks.

During the colonoscopies, cold snare polypectomies (CSP) were conducted for polyps and excisional biopsy forceps were considered as a second‐line option with polyps of size ≤3 mm which is technically difficult for CSP.^24^, ^25^ Only fresh mucosal tissues with polyps <1 cm in size were included. To train the model to learn advanced pathological features, biopsies from advanced neoplasms were also obtained. Once the polypectomy or biopsy of the lesion was completed, the specimen was immediately imaged with a hyperspectral (HS) camera, according to our standard operational procedures (Data S1). A brief workflow is shown in Figure 1A.

### 2.2 Experiment equipment and acquisition of HS images

A GaiaMicro‐V10E‐HR model machine (Double Synthesis, Beijing, China) was used, comprising a GaiaMicro series HS camera, microscope, calibration whiteboard, system holder, and microscope objective. Gaia Micro series contain built‐in push‐broom HS cameras that push space to obtain HS cubes. Images were collected with a spatial size of 1101 lines, producing 960 × 1101 × 360 HS cubes (number of lines × number of rows × number of bands).

### 2.3 Histopathological examination

After formalin‐fixed and paraffin‐embedded, tissues were sectioned and placed into glass slides, and finally stained with hematoxylin and eosin. Two experienced pathologists (with over 5 years of reading experience) independently examined all tissue slides and made the final diagnosis according to the World Health Organization classification for colorectal tumors.^26^ Serrated lesions with a low incidence were not included in this pilot study.

Tissue samples were pathologically categorized as neoplastic or nonneoplastic lesions according to the consensus of the US multi‐society task force on CRC.^27^ NNP were referred to as hyperplastic polyps (HP) and inflammatory polyps. Neoplastic lesions including non‐advanced adenoma (NAA, histologically diagnosed as tubular adenoma with low‐grade dysplasia) and advanced neoplasia (AN, histologically diagnosed with advanced histologic features of high‐grade dysplasia, villous change, or invasive carcinoma).

### 2.4 Spectral analysis and image processing

Reflectance calibration was performed to convert the original light intensity into reflectivity and represent the light intensity information. Data standardization processing was conducted to eliminate the dimensional influences and address the comparability between metrics, accelerating the convergence rate and improving the accuracy of the model. Details are provided in Data S1.

To balance the data with different histological labels in the model, we performed data augmentation for each group by randomly selecting HS images from different fields of view for each sample. To address the challenges posed by the high dimensionality of HS images, two‐dimensional (2D) images were generated using the spectral and spatial dimension information extracted from the original HS images. The intuitive forms of the one‐dimensional and 2D image data are shown in Figure 1B. Nine hundred 2D images were generated from each selected HS image through dimension reduction. Data on the tissues and HS images are shown in Table 1.

**TABLE 1:** Number of tissue samples and hyperspectral images.

	NNP	NAA	AN	Total
Tissue samples	28	53	13	94
Selected hyperspectral images	56	53	52	161
Two‐dimensional Images	56 × 900	53 × 900	52 × 900	144,900

Abbreviations: AN, advanced neoplasia; NNP, nonneoplastic polyp; NAA, non‐advanced adenoma.

### 2.5 Development of residual network model and transfer learning

A 2D convolutional neural network was trained for the automatic classification of colorectal lesion patches. The network was able to combine the spectral and spatial information of the specimens. We developed a residual network (ResNet) to combat the vanishing gradient and network degradation caused by the deepening of the classic convolutional neural network and improve performance.^28^

Transfer learning is a method allowing the fine‐tuning of existing model algorithms, making them applicable to new fields or features.^29^ The transfer learning method adopted in our study loaded the weights of the pretrained ResNet model, added a fully connected layer after the original ResNet model, and re‐established all layers of the network to improve the recognition effect of different case combinations (Figure 1B).

### 2.6 Assessment of model and the assistant efficiency for novices

The diagnostic performance of endoscopists for small and diminutive colorectal polyps was evaluated by four expert (>2000 screening colonoscopies) and six novice endoscopists (200–400 colonoscopies) using white‐light images of lesions in the test set. An endoscopist test was performed using a questionnaire. Information regarding the images and patients in the test set is presented in Table 2.

**TABLE 2:** The information of selected testing images and patients/lesions.

Image	Dataset A	Dataset B	Dataset C
Image type	White light endoscopic image	Hyperspectral image	2‐D images from HSI
Image content	56 images of 32 NAA, 8 AN and 16 NNP	48 images of 16 NAA, 16 AN and 16 NNP	43,200 images of 14,400 NAA, 14400 AN and 14,400 NNP
Process	Selected according to inclusion and exclusion criteria		Processing dataset B
Patient content	28 cases of 16 NAA, 4 AN and 8 NNP	28 cases of 16 NAA, 4 AN and 8 NNP	28 cases of 16 NAA, 4 AN and 8 NNP

*Note*: Details of patients and lesions are shown in Data S1.

Abbreviations: AN, advanced neoplasia; HSI, hyperspectral imaging; NAA, non‐advanced adenoma; NNP, nonneoplastic polyp.

To evaluate the assistant efficiency of the novice and expert endoscopists, they were asked to review the images twice. Endoscopists were tested independently first, then they were informed of the HSI‐AI model's prediction before they made their final diagnosis, referred to as the HSI‐AI‐assisted diagnosis test. The two tests were separated by a 2‐week washout period, and the final results were compared.

### 2.7 Outcome measures and statistical analysis

The primary outcome measure is the diagnostic performance of the model, which was indicated by a confusion matrix. Accuracy represents the correct result proportion of the classification model in the total observed value. Sensitivity, specificity, positive predictive value, NPV, receiver operating characteristic (ROC) curve analysis and the area under the ROC curve (AUC) measurement were used to assess model performance.

The secondary outcome included the diagnostic performances of the novice and expert endoscopists, and the assistant efficiency of the HSIAI model. Various indexes of all endoscopists are presented as the median and interquartile range (IQR). The Mann–Whitney *U* test was used to compare the diagnostic performance between independent endoscopists and HSI‐AI assisted endoscopists.^30^, ^31^ All statistical analyses were performed by R software 4.1.0 (https://www.r‐project.org/). The two‐sided *p*‐value less than .05 was considered statistically significant in all calculations.

## 3 RESULTS

### 3.1 Patients and histopathology

Overall, 116 fresh specimens from 86 patients who underwent a colonoscopy and polypectomy or biopsy between October and December 2022 were examined. Twenty‐two samples were excluded owing to the diagnosis of inflammatory bowel disease, neuroendocrine tumors, or other pathological types.

Finally, 94 tissues (64 polypectomies and 30 biopsies) from 73 patients were included. Upon histopathological assessment, 53 patients were diagnosed with tubular adenomas with low‐grade dysplasia (NAA), 28 with NNP, and 13 with AN (six tubular adenomas with high‐grade dysplasia, three villous adenomas, and four adenocarcinomas). After data augmentation, 144,900 2D images generated from 161 HS images were grouped into training, test, and validation sets at a ratio of 6:3:1. The specific sample inclusion and data allocation process were shown in Data S1.

### 3.2 Original spectral analysis

By scrutinizing the typical spectra of different tissues, the shape and peak size of the spectral curves of the three different tissue structures were found to be clearly different with band length from 406.9 to 662.1 nm, providing a good theoretical basis for the model study. As shown in Data S1, the reflection rates of NNP, NAA and AN were significantly different when the band lengths were 575 nm (*p* < 0.001).

### 3.3 Preexperiment of histological prediction

To verify the reliability of the data and explore the correlation in the 2D image data, two quantification models were performed in batches, and the results of the second model were considered as our final reliable results.

**FIGURE 2:**
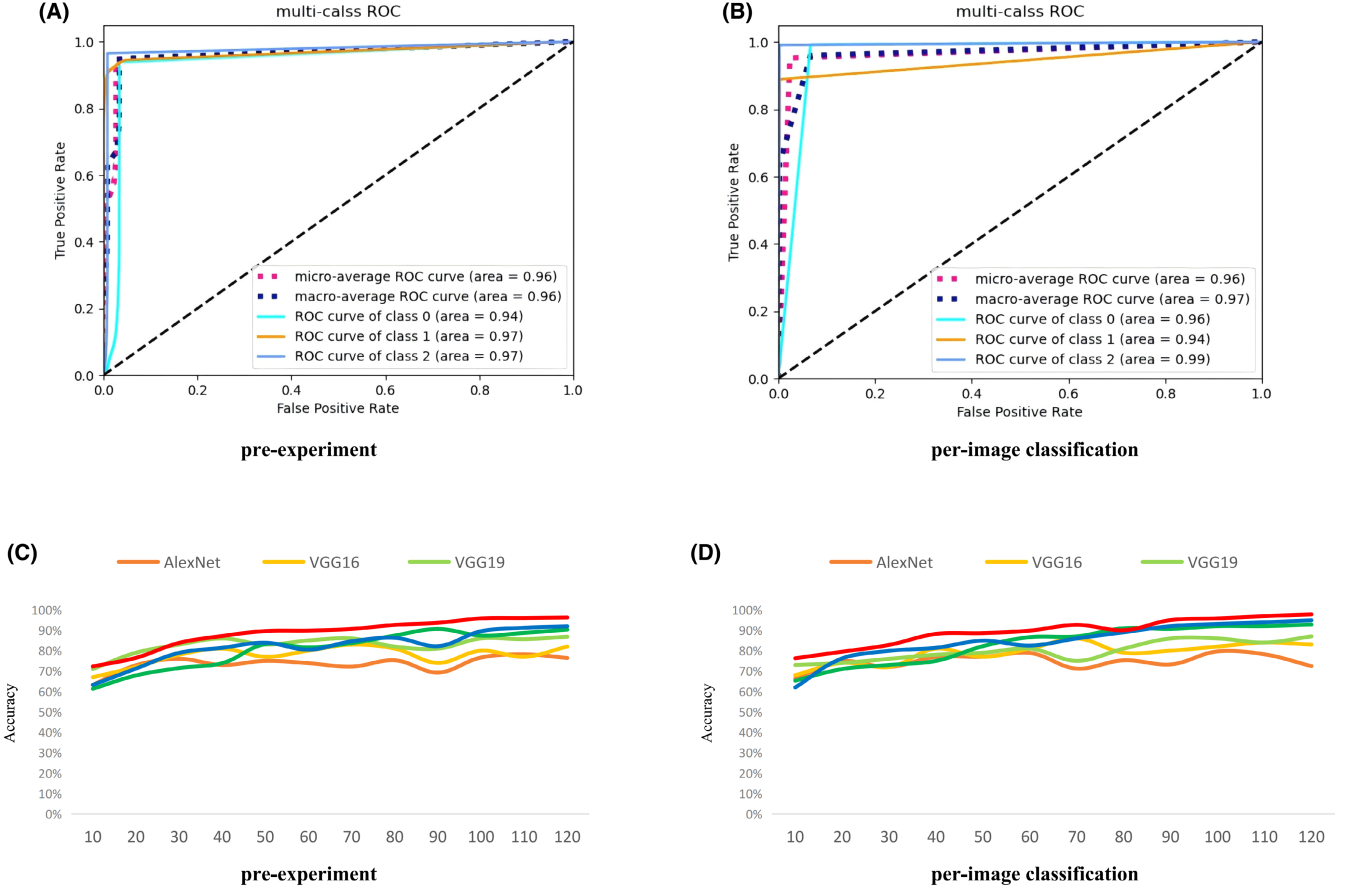
Automated diagnosis with residual neural network on hyperspectral images. (A, B) Training validation and test results of the neural network for the classification of nonneoplastic polyp/non‐advanced adenoma/advanced neoplasia. (C, D) comparison of variable neural network models for automated classification.

In the first model, the 2D images were programmed to be shuffled randomly regardless of the individual heterogeneity of each specimen. The results showed a high accuracy for each group, with an AUC of 0.97 for the training set and 0.96 for the test set, which indicated a certain correlation between the different images from each tissue specimen and the consistency of pixels of each original HS image (Figure 2A).

### 3.4 Histological prediction with per‐image and per‐patient classification

The second model was applied to independent patients in the test set. The datasets were grouped individually according to the specimen, and the sequence of images was shuffled in each dataset. 2D images in each dataset were generated from the original HS images of each specimen, and the diagnostic performance (individual prediction) was tested. The prediction was deemed correct when more than 80% of the 2D images generated from the sample (original HS image) were classified correctly into the corresponding category.

For the model that classified the diagnosis (NNP, NAA, or AN), the test results and ROC curves showed good performance (Figure 2B), with an accuracy of 95.8% and a macro‐AUC of 0.97. The diagnostic performance of the per‐image model for histological predictions is shown in Table 3. The model showed great potential for the classification of neoplastic lesions or not, with a sensitivity and specificity of neoplastic lesion (including NAA and AN) of 99.0% and 96.0%, respectively. Confusion matrix of the models is shown in Data S1.

**TABLE 3:** Diagnostic performance of HSIAI model and endoscopists.

Parameter	NNP	HSIAI model with 2‐D images	
		NAA	AN
AUC	0.96	0.94	0.99
Sensitivity (95% CI)	96.0 (95.7–96.3)	94.0 (93.6–94.4)	99.0 (98.8–99.2)
Specificity (95% CI)	99.0 (98.9–99.1)	99.0 (98.9–99.1)	96.5 (96.3–96.7)
PPV (95% CI)	98.0 (97.7–98.1)	93.3 (66.0–99.7)	93.4 (93.0–93.7)
NPV (95% CI)	98.0 (97.9–98.2)	97.1 (96.9–97.2)	99.5 (99.4–99.6)

		Endoscopists with white light endoscopic images		
		Neoplastic polyp		
	Experts (*n* = 4)^a^	Novices (*n* = 6)^a^	HSI‐assistant Experts^a^	HSI‐assistant novices^a^
Accuracy	91.1 [87.5, 93.8]	71.4 [66.1, 79.4]	94.7 [92.0, 96.4]	94.7 [92.9, 96.4] **
Sensitivity	92.5 [90.0, 96.3]	72.5 [70.0, 86.3]	97.5 [95.0, 100.0]	95.0 [95.0, 98.8] *
Specificity	87.5 [81.3, 87.5]	62.5 [62.5, 71.9]	87.5 [84.4, 87.5]	87.5 [87.5, 96.9] *
PPV	94.9 [92.5, 95.1]	84.5 [80.8, 87.1]	95.1 [93.9, 95.2]	95.2 [95.1, 98.8]**
NPV	82.7 [76.2, 90.6]	50.0 [42.5, 66.1]	93.75 [87.1, 100.0]	88.2 [86.2, 97.2] **

Abbreviations: AN, advanced neoplasia; AUC, the area under the curve; CI, confidence interval; NAA, non‐advanced adenoma; NNP, nonneoplastic polyp; NPV, negative predictive value; PPV, positive predictive value.

^a^ The data was present as median (interquartile range); *p<0.05, **p <0.01.

### 3.5 Assistant efficiency of the AI model for endoscopists

White‐light endoscopic images of lesions in the test set were reviewed by endoscopists to assess the performance of endoscopic distinction between NNP and neoplastic polyps. Neoplastic polyps were diagnosed with a median per‐image accuracy of 91.1% by the experienced endoscopists and 71.4% by the novices. In the HSIAI model, each neoplastic polyp was identified from NNP in the HS images in the test set and only one NAA was misclassified as AN.

**FIGURE 3:**
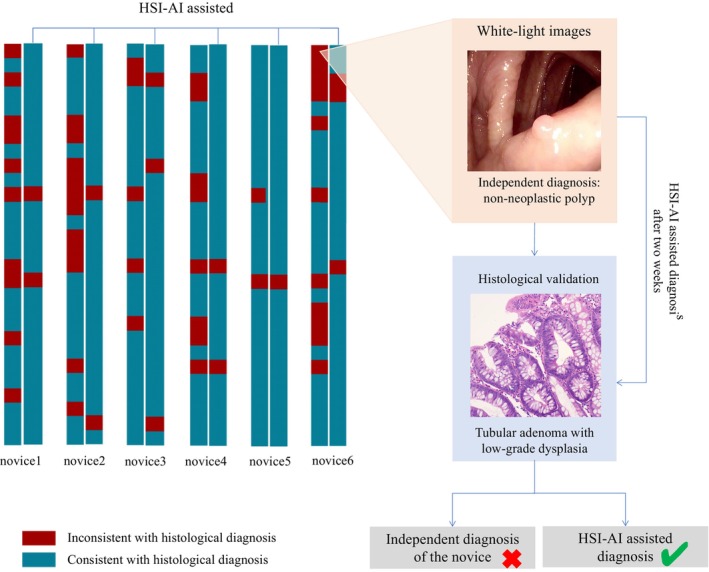
Diagnostic performance of novices with HSI‐AI assistance compared without HSI‐AI assistance in identifying neoplastic and nonneoplastic colorectal polyps by using white‐light endoscopic images.

With the assistance of the HSIAI model, novices achieved an median accuracy of 94.7%, which was nearly comparable to that of experts. In novice endoscopists, HSIAI assisted diagnosis was significantly different from independent diagnosis (*p* < 0.01), while there was no difference among experts (*p* = 0.63). The NPV of the several novices exceeded 90%, meeting the requirements of PIVI for resect‐and‐discard strategy, and the overall median NPV of novices increased from 50.0% to 88.2% (*p* = 0.013). Various diagnostic indices of the novice endoscopists were further improved with the assistance of the HSIAI model (Table 3). Details for novices are presented in Figure 3.

## 4 DISCUSSION

Here, we present an HSI system combined with a machine‐learning approach for the histological prediction of colorectal polyps. According to our study, HSI could precisely distinguish over 90% of colorectal neoplastic polyps from NNP and identify advanced neoplasia with high‐risk histological features based on fresh tissues. With the assistance of the HSI, the median NPV in novices increased from 50.0% to 88.2% (*p* = 0.013), which was expected to meet the PIVI criteria. The HSI system has the potential to differentiate neoplastic colorectal polyps, which may reduce the burden of pathological evaluation and facilitate the application of resect‐and‐discard strategies to improve patient outcomes.

A precise HSI diagnostic model for colorectal polyps was developed based on clinical issues and was evaluated using clinical criteria. Accurate endoscopic prediction of histology of small and diminutive polyps renders the “resect‐and‐discard strategy” possible instead of unnecessary polypectomies and histopathologic assessment, reducing the excessive cost. Despite the development of several advanced optical techniques to improve accuracy, training novices to become proficient in these techniques can be time‐ and cost‐consuming, limiting their widespread adoption.

Ultimately, the overall diagnostic accuracy of model for classifying NNP/NAA/AN was 95.8%, which was comparable to or even higher than that reported in previous studies. A CADx developed by Quirine et al. (2021) showed the high accuracies of 88.3% for high‐definition white‐light, 86.7% for blue‐light imaging (BLI), and 95.0% for combined multimodal imaging, while it did not reach an NPV of ≥90%.^32^ A more recent CAD developed with BLI by Rondonotti et al. (2023) revealed that AI‐assisted diagnosis accuracy was up to PIVI benchmark for experts (91.9%, 95 %CI 88.5%‐94.5%) while still low for novices (82.3%, 95 % CI 76.4%–87.3%).^33^ More CADx diagnosis systems were developed using NBI and endocytoscopy, of which Michael et al. (2019) validated with unaltered videos of standard colonoscopy with the accuracy of 94% (95% CI 86%–97%).^11^ However, 15% of polyps in the test set did not conform with sufficient confidence, with 83% specificity. In the present study, the HSI‐AI model successfully predicted neoplastic lesions with an NPV of up to 98.0% (95% CI: 97.7%–98.1%), reaching the ASGE‐PIVI benchmark for clinical use of optical diagnosis. In addition, advanced neoplasia was included to improve the capability of the model for the early differential diagnosis of CRC and surveillance interval determination.

Endoscopic prediction of polyp histology is highly subject to endoscopist dependence. Recent studies have also shown that the performance of fully trained expert endoscopists meets the PIVI criteria, whereas the same is not true in community endoscopy practices.^14^ The median accuracy of neoplastic polyps was 71.4% for novices, similar to that in the present study. With the assistance of the HSIAI model, the accuracy significantly improved to 94.7%, comparable to that of experts. The NPV increased with the assistance of HSI from 50.0% to 88.2% (*p* = 0.013) in novices, which was also up to the standard of PIVI. These results indicate that the assistance of HSIAI model can improve the diagnostic performance of novices, but has limited benefit for experts, likely because experts already have a high accuracy in diagnosing neoplastic polyps.

To verify the accuracy of the HSI model, the classification results were validated by histological diagnosis of polyp tissues. The prediction results of the HSI model were highly consistent with those of the pathological diagnosis, with an AUC of 0.97. Only NAA were misdiagnosed, which may be due to variable lesion surface features and patient heterogeneity. Multiple histological features can exist in different fields of one lesion, resulting in different spectral information in the 2D images. Therefore, a better standard should be explored with a larger sample size to eliminate the heterogeneity of 2D images from tissues and to represent the final classification of the lesion.

This study has several strengths. First, HSI techniques were used to differentiate adenomatous and nonneoplastic colorectal polyps. Although a few previous studies have explored the application of HSI for CRC, the majority of them based on hematoxylin and eosin‐stained tissue sections that needed slide preparation.^34^, ^35^ Other studies using surgical specimens from surgical resection,^36^, ^37^, ^38^ which mainly distinguished advanced carcinoma from normal tissue, neglected the diagnosis of precancerous lesions and early cancer. Though some in vivo studies of HS endoscopy have been conducted, their methodological limitations or small sample sizes resulted in limited reliability and generalizability of the findings.^39^, ^40^ Second, although variable models have been developed to improve the performance, and ResNet is relatively robust and accurate by introducing the transfer learning method, the overfitting phenomenon was significantly reduced and the accuracy was further improved (Figure 2D, by interpatient classification). Third, experts and novices conducted an optical diagnosis of colorectal polyps, and the efficiency of HSI assistance for novices was assessed. With HSI assistance, the novices were expected to meet the PIVI criteria.

However, the study also had several limitations. Firstly, as a pilot study, the sample size was limited owing to the low incidence of advanced neoplasia in small polyps. A large prospective study is required to enlarge the training database and validate the model by incorporating more HS data on normal and neoplastic tissues from a variety of anatomical sites and patients. Secondly, HS images were obtained only in vitro using fresh tissues from polypectomies or excisional biopsies. Additionally, serrated lesions with a low incidence were not included in our study, which would allow for the feasibility of HS diagnosis in the future to be explored.

## 5 CONCLUSION

In conclusion, our study demonstrated that HSI can achieve high accuracy in sorting colorectal polyps into adenomas versus NNP and even identify advanced neoplasia based on fresh tissues from a colon tissue. The tool could improve the diagnostic performance of novices and show the potential of AI‐assisted diagnosis synchronized with colonoscopy. We plan to evaluate the HSI‐assisted resect‐and‐discard strategy in day‐to‐day practice for CRC screening program and develop HS endoscopy for in vivo studies.

## AUTHOR CONTRIBUTIONS

**Cheng Peng:** Conceptualization (lead); data curation (equal); formal analysis (equal); investigation (equal); methodology (equal); project administration (equal); resources (equal); software (equal); validation (equal); writing – original draft (lead); writing – review and editing (equal). **Chong Xuan Tian:** Conceptualization (equal); data curation (equal); formal analysis (equal); methodology (equal); software (equal); writing – original draft (equal); writing – review and editing (equal). **Yijun Mu:** Conceptualization (supporting); data curation (supporting); investigation (supporting); resources (supporting); validation (supporting); visualization (supporting); writing – review and editing (equal). **Mingjun Ma:** Conceptualization (supporting); data curation (supporting); investigation (supporting); methodology (supporting); resources (supporting); supervision (supporting); validation (equal); visualization (equal); writing – review and editing (equal). **Zhenlei Zhang:** Formal analysis (equal); investigation (equal); methodology (equal); resources (equal); software (equal); validation (equal); writing – review and editing (supporting). **Meng Wan:** Formal analysis (supporting); investigation (supporting); methodology (supporting); resources (supporting); supervision (equal); validation (equal); visualization (equal); writing – review and editing (equal). **Jing Liu:** Data curation (supporting); formal analysis (supporting); investigation (supporting); methodology (supporting); resources (supporting); supervision (supporting); validation (equal); visualization (equal); writing – review and editing (equal). **Zhen Li:** Conceptualization (supporting); formal analysis (supporting); funding acquisition (supporting); methodology (supporting); project administration (supporting); supervision (equal); validation (equal); visualization (equal); writing – review and editing (equal). **Xiuli Zuo:** Conceptualization (equal); data curation (supporting); funding acquisition (equal); investigation (equal); project administration (supporting); resources (equal); supervision (equal); writing – review and editing (equal). **Wei Li:** Conceptualization (supporting); funding acquisition (equal); methodology (equal); project administration (equal); software (equal); supervision (equal); visualization (equal); writing – review and editing (equal). **Yanqing Li:** Conceptualization (supporting); funding acquisition (lead); methodology (supporting); project administration (equal); resources (equal); supervision (lead); writing – review and editing (equal).

## FUNDING INFORMATION

This work was supported by the Shandong Provincial Key Research and Development Program (Major Scientific and Technological Innovation Project) (2021CXGC010506), National Key R&D Program of China (2019YFE0117800), National Natural Science Foundation of China (26010105132243) and National Natural Science Foundation of China (22176115).

## CONFLICT OF INTEREST STATEMENT

Authors declare no Conflict of Interest for this article.

## ETHICS STATEMENT

The study protocol was approved by the Medical Ethics Committee of Qilu Hospital of Shandong University (Reference no. 2022(026)).

## CONSENT

Informed consent was obtained from all participants/patients.

## Supporting information


Data S1.

## Data Availability

The datasets used and/or analyzed during the current study are available from the corresponding author on reasonable request.
