# Supplementary material for: Hyperspectral imaging facilitating resect‐and‐discard strategy through artificial intelligence‐assisted diagnosis of colorectal polyps: A pilot study
Source: Cancer Med. 2024 Sep 25;13(18):e70195. doi: 10.1002/cam4.70195 (PMC11423483; doi:10.1002/cam4.70195)
Supplement: Supplementary file 1 — Data S1. [file CAM4-13-e70195-s001.zip › Data S1/supplementary material 1.docx]

**Supplementary material 1** Standard operational procedures (SOPs) of specimen preparation and hyperspectral images acquisition.

To ensure high quality and efficient image acquisition, pre-experiments were conducted and standard operational procedures (SOPs) was developed. The SOPs included optimal time of exposure, a dynamic range configuration, an appropriate calibration and focusing procedure, and the pick-and place process for fresh tissue.

With the initial adjustment of focal length and acquisition parameters set, the tissue was gently lifted with disposable tweezers and placed on the slide on the platform of microscope once after the biopsy. Blotting paper was used to absorb the surface water of the tissue in order to avoid the impact of water on refraction and imaging. Adjusted the fine quasi-focal helix to clear the field of view at low magnification (5x) and then pulled out the lever on the microscope to preview focus. After clearness of hyperspectral image was determined by looking at the curve sharpness of the test object, hyperspectral image acquisition was performed. Each specimen was imaged at least 2 fields to reduce the failure rate. The HS system is a spectral imaging system based on a charge-coupled device detector. This system operates in the visual and near-infrared spectral range of 400–1000 nm with a spectral resolution of 2.8 nm, sampling 360 spectral channels, and 900 spatial pixels. Images was collected with a spatial size of 1101 lines, producing HS cubes of 960 × 1101 × 360, i.e., number of lines × number of rows × number of bands.

In order to avoid the excessive exposure time resulting in tissue water loss which may affect the pathological examination of the tissue, no more than three minutes were required the time of exposure to air.
