# Supplementary material for: Hyperspectral imaging facilitating resect‐and‐discard strategy through artificial intelligence‐assisted diagnosis of colorectal polyps: A pilot study
Source: Cancer Med. 2024 Sep 25;13(18):e70195. doi: 10.1002/cam4.70195 (PMC11423483; doi:10.1002/cam4.70195)
Supplement: Supplementary file 1 — Data S1. [file CAM4-13-e70195-s001.zip › Data S1/supplementary material 2.pdf]

Supplementary material 2

Spectral analysis and image processing.

Spectral analysis

In the process of hyperspectral imaging, a dark current is usually generated by the interference of environmental light, system power supply and other factors. Dark currents are temperature dependent and proportional to the integration time. Therefore, to convert the original light intensity into reflectivity, reference and dark images are also required when collecting tissue images. In this study, the reference image was taken with a standard reflection surface placed in the scene, dark current was measured by keeping the camera shutter closed, and the standard reflection whiteboard provided absolute reflection ratio values (provided by the National Institute of Metrology). The raw data were then corrected using the following equations:

$$P_{ref} = \frac{P_{raw} - P_{dark}}{P_{white} - P_{dark}} \qquad (1)$$

where P_{ref} is the calculated reflectivity value, P_{raw} is the original data emissivity value of a given pixel, P_{dark} is the dark current intensity of a given pixel, and P_{white} is the white board reference intensity.

The HS image before the reflectance calibration is shown in Fig.S1a, and the spectral curve in the spectrum map represents the light intensity information, which has no practical significance. After the reflectance calibration, the HS image is presented as Fig.S1b, with the hyperspectral reflectivity curve reflecting the light reflection of different tissues on its right.

Fig.S1 Reflectance calibration of hyperspectral images

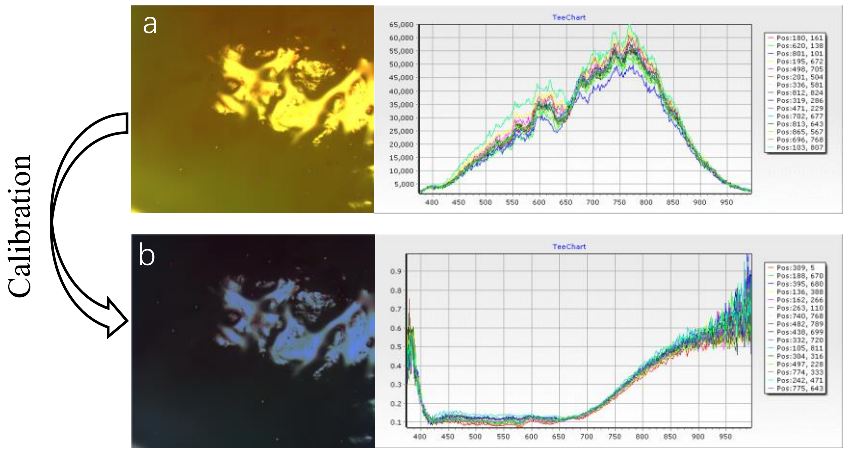

To eliminate dimensional influences between metrics, data standardization processing is required to address comparability between data metrics. This process is crucial for HS images with multidimensional information, which can accelerate the convergence rate of the model and improve the accuracy of the model after standardizing the original data. This experiment adopts linear function standardization, also known as deviation standardization, which is a linear transformation of the original data, in which the resulting value is mapped between [0,1], and the conversion function is:

$$I_{nom} = \frac{I - I_{min}}{I_{max} - I_{min}} \quad (2)$$

where I_{nom} is standardized data, x is original data, I_{max} is the maximum value of sample data, and I_{min} is the minimum value of the sample data. After the original data was standardized, each index was in the same order of magnitude, reducing the image of the results of data analysis.

Image processing and Two-dimensional image generation

To address the challenges imposed by the high dimensionality of the HS images, two-dimensional images was generated with the spectral dimension and spatial dimension information extracted from the original HS images. Interested pixels of each hyperspectral image were selected by means of interval sampling. With the diverse absorbing peaks tiled, the spectral information carried by each interested pixel was processed and spliced together into a two-dimensional image. 900 two-dimensional images were generated for each original hyperspectral image.

The two-dimensional image at this stage contained not only the spectral information of the one-dimensional data but also the spectral structural information of the nearby pixels in the two-dimensional image. Intuitive forms of one-dimensional data and two-dimensional image data are shown in Fig.1b.
