# Supplementary material for: Hyperspectral imaging facilitating resect‐and‐discard strategy through artificial intelligence‐assisted diagnosis of colorectal polyps: A pilot study
Source: Cancer Med. 2024 Sep 25;13(18):e70195. doi: 10.1002/cam4.70195 (PMC11423483; doi:10.1002/cam4.70195)
Supplement: Supplementary file 1 — Data S1. [file CAM4-13-e70195-s001.zip › Data S1/supplementary material 3.docx]

**Supplementary material 3** Supplement information of the testing set.

| Patient information | n = 28 |
| --- | --- |
| Age, mean (SD) | 46.9 (12.6) |
| Gender, n (%) |  |
| male | 18 (64.3) |
| female | 10 (35.7) |
| Polyp characteristics | n = 28 |
| Location, n (%) |  |
| cecum | 1 (3.6) |
| ascending colon | 4 (14.3) |
| transcending colon | 6 (21.4) |
| descending colon | 7 (25.0) |
| sigmoid colon | 6 (21.4) |
| rectum | 4 (14.3) |
| Size, n (%) |  |
| ≤ 5mm | 18 (64.3) |
| > 5mm | 10 (35.7) |
| Histology, n (%) |  |
| Hyperplastic or inflammatory polyp | 8 (28.6) |
| Neoplastic polyp | 20 (71.4) |
