# Supplementary material for: Hyperspectral imaging facilitating resect‐and‐discard strategy through artificial intelligence‐assisted diagnosis of colorectal polyps: A pilot study
Source: Cancer Med. 2024 Sep 25;13(18):e70195. doi: 10.1002/cam4.70195 (PMC11423483; doi:10.1002/cam4.70195)
Supplement: Supplementary file 1 — Data S1. [file CAM4-13-e70195-s001.zip › Data S1/supplementary material 5.pdf]

Supplementary material 5

Quantitative analysis and comparison of spectral signals.

After reflectance calibration, the spectral curves of the three types of tissues showed certain differences. As shown in Fig 1, differences were more prominent in the 500-575nm band range. On the whole, in the range of 500-575nm, the reflection curve of NNP showed a downward trend, while that of NAA showed a trend of first rising and then declining, and the curve of AN showed a slow rising trend.

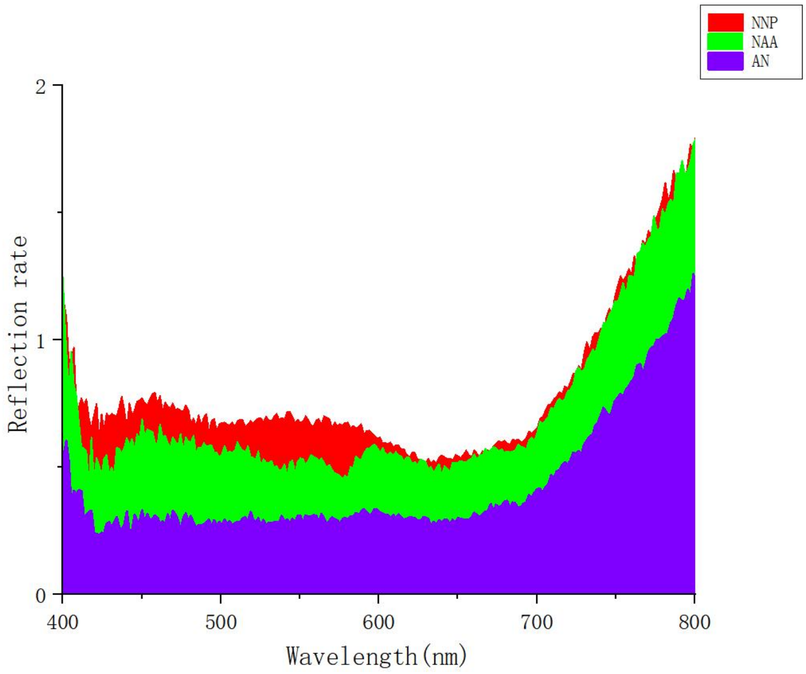

Fig1. Spectral curves after reflectance calibration

However, the fluctuation of spectral curves of different tissues is obvious. In order to reduce noise interference, Savitzky-Golay filter was used to achieve curve smoothing. The advantage of Savitzky-Golay filter is that it can retain signal change information more effectively while filtering smooth. To represent the range of characteristic bands of the three types of organizations more intuitively, tangential lines were drawn at each point (**Fig.2**). However, at 406.9mm band and 662.1nm band, the tangent slopes

of the three groups were almost equal (**Table 1** for details). In the range of 406.9-662.1nm, the tangent slopes of the three are the most different. Combined with the actual false-color images, we find that there is no spatial information before 406.9nm and after 662.1nm, which proves that the critical point where the curves of the three are significantly different is at 406.9nm and 662.1nm.

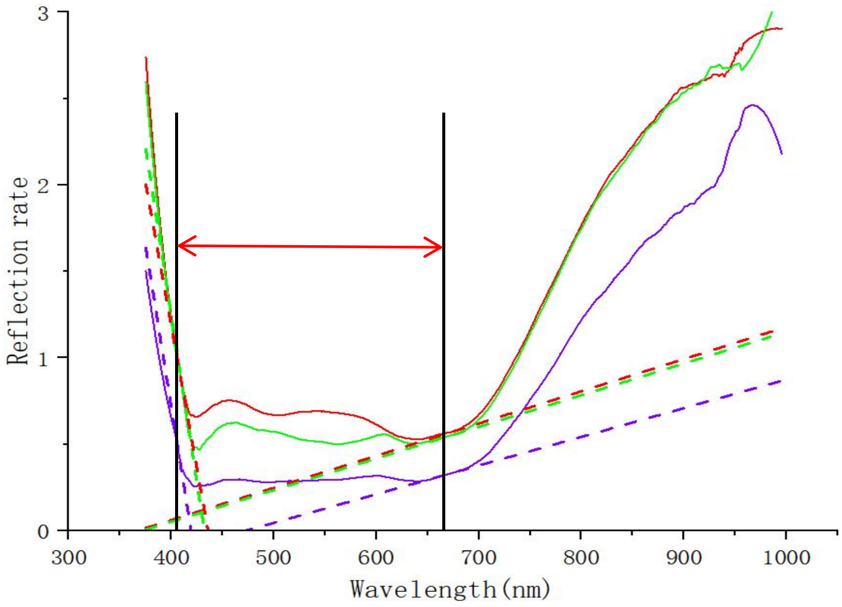

Fig 2. Spectral curves and tangential lines

Color of tangential line	Coordinate	Angle
R	(406.9,0.98349)	177.832°
G	(406.9,0.98832)	177.815°
B	(406.9,0.46326)	177.827°
R	(662.1,0.55483)	0.10492°
G	(662.1,0.54364)	0.10489°
B	(662.1,0.31449)	0.10488°

Table 1.Coordinates and angle information of critical points

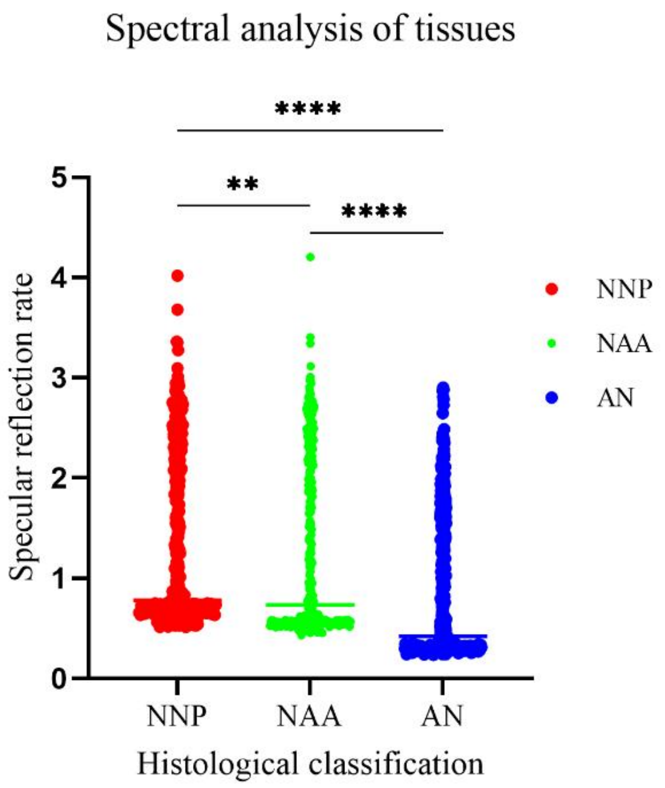

Fig 3. Quantitative analysis of spectral signals at band length of 575nm.

Average spectral signals of each tissue types was calculated as reference signals: non-neoplastic polyp (red), non-advanced adenoma (green), and advanced neoplasm (blue). Vertical lines show mean value and SD. Statistical analysis was performed using Kruskal-Wallis test for multiple comparisons. **indicates $p<0.01$ and ****indicates $p<0.0001$.
