# Supplementary material for: Hyperspectral imaging facilitating resect‐and‐discard strategy through artificial intelligence‐assisted diagnosis of colorectal polyps: A pilot study
Source: Cancer Med. 2024 Sep 25;13(18):e70195. doi: 10.1002/cam4.70195 (PMC11423483; doi:10.1002/cam4.70195)
Supplement: Supplementary file 1 — Data S1. [file CAM4-13-e70195-s001.zip › Data S1/supplementary material 6.pdf]

Supplementary material 6

Confusion matrix of the three-subtype classification.

Pre-experiment

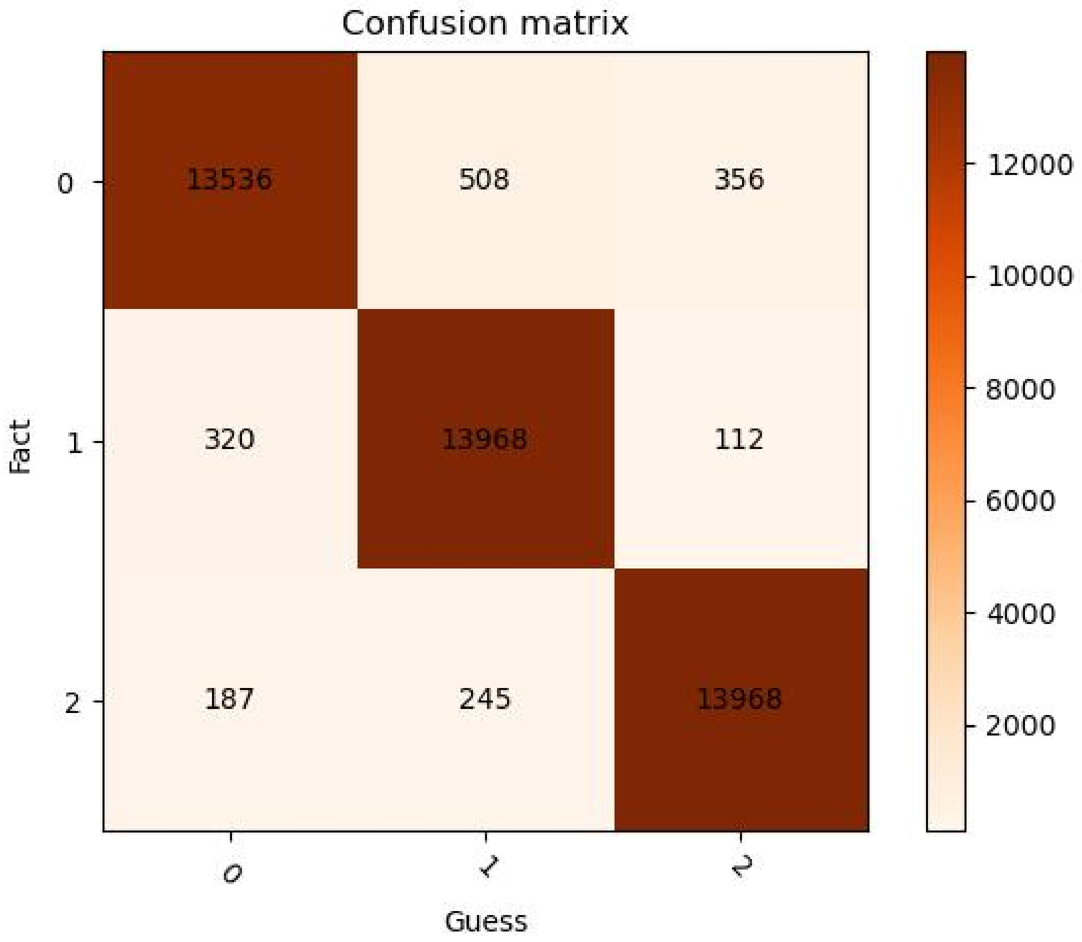

Confusion matrix of the classification of non-neoplastic polyp/non-advanced adenoma/advanced neoplasia

0 represents non-neoplastic polyp;
1 represents non-advanced adenoma;
2 represents advanced neoplasia.

Per-image classification

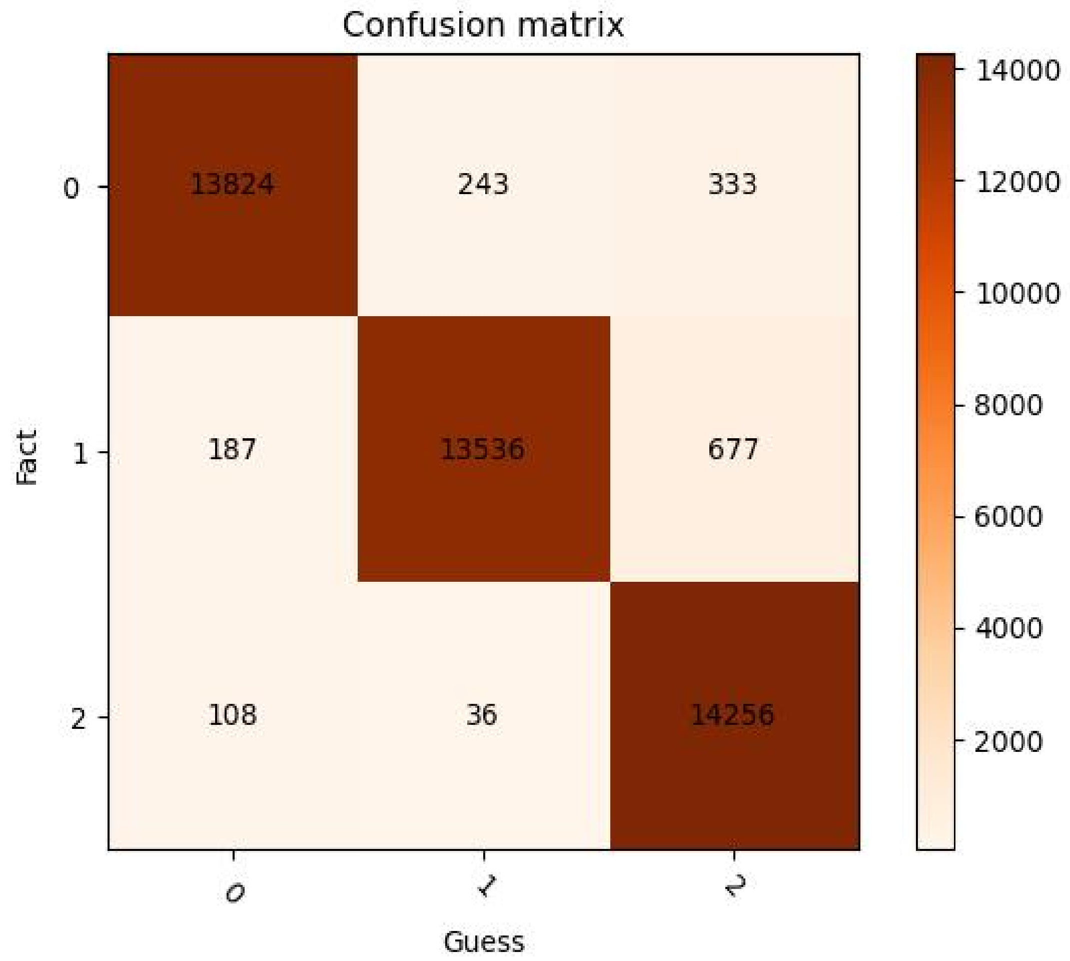

Per-patient classification

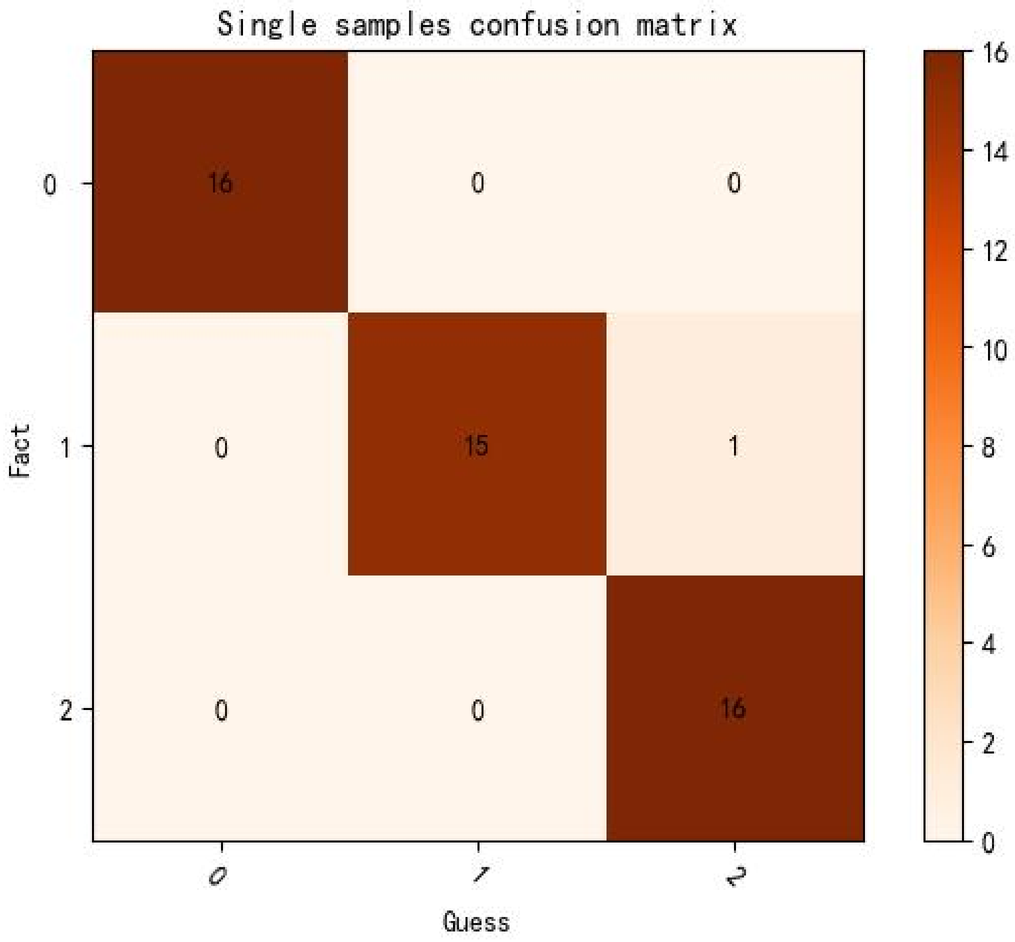

Confusion matrix for classification of non-neoplastic polyp/non-advanced adenoma/advanced neoplasia

0 represents non-neoplastic polyp;
1 represents non-advanced adenoma;
2 represents advanced neoplasia.
